# Tamarind (*Tamarindus indica* L.) Components as a Sustainable Replacement for Pork Meat in Frankfurter Sausages

**DOI:** 10.3390/foods14020197

**Published:** 2025-01-10

**Authors:** Rafael Sepúlveda F. Trevisan Passos, Camila Cristina A. de Sousa, Mauricio C. A. da Silva, Ana M. Herrero, Claudia Ruiz-Capillas, Carlos Pasqualin Cavalheiro

**Affiliations:** 1 Laboratório de Indústria e Inspeção de Carnes e Derivados, Universidade Federal da Bahia, Salvador 40170-115, Brazil; rafael.sepulveda@ufba.br (R.S.F.T.P.); camila.cristina@ufba.br (C.C.A.d.S.); mcasilva@ufba.br (M.C.A.d.S.); 2 INDMEAT Group, Department of Meat and Fish Products, Institute of Food Science, Technology and Nutrition (ICTAN-CSIC), 28040 Madrid, Spain; ana.herrero@ictan.csic.es

**Keywords:** meat products, by-products, pulp, peel, seed, technological properties

## Abstract

Five types of frankfurters were formulated: a control without tamarind (T0) and four samples using 5% tamarind pulp paste (PT5), seeds (ST5), peel (CT5), and a blend of all of them (PSCT5), replacing the same portion of meat. The inclusion of tamarind components led to a reduction in the moisture and protein content of the reformulated frankfurters. In terms of mineral composition, CT5 showed the highest (*p* < 0.05) calcium content. Additionally, ST5 and CT5 treatments demonstrated the lowest processing loss values. The pH was lower in the PT5 treatment (*p* < 0.05). Incorporating tamarind components reduced the lightness (L*) of the frankfurters, resulting in darker sausages. However, ST5 exhibited greater redness (a*), while higher yellowness (b*) values were observed in PT5 and CT5 treatments (*p* < 0.05). Texture analysis revealed no differences (*p* > 0.05) in hardness and chewiness between T0 and PT5; however, ST5 exhibited the highest values for these parameters (*p* < 0.05). No variation in the conformational order of the lipid acyl chains due to the incorporation of tamarind compounds was observed related to physical entrapment of these compounds in the frankfurter matrix. Both T0 and PT5 were well accepted by consumers, and scores higher than 7 were observed for overall acceptability and purchase intention. The study demonstrated that incorporating tamarind components, especially pulp paste (PT), is a viable alternative for replacing lean pork meat in frankfurters, improving the sustainable aspects of frankfurter production.

## 1. Introduction

Meat is a high-quality nutritional food, recommended for its contribution to the intake of essential nutrients, including high-quality protein, essential fatty acids, vitamin B12, and minerals such as iron and zinc [1]. Over the past 20 years, global meat consumption has increased significantly, rising by 58% [2]. Moreover, processed meat products such as frankfurters, patties, meatballs, and fresh sausages are particularly favored by consumers due to their affordability and convenience in preparation.

Frankfurters are cooked sausages that are among the most widely consumed worldwide. Their popularity stems from their ease of preparation, versatility, appealing sensory characteristics, and affordability. These sausages are enjoyed by people of all ages, whether at home or in fast-food restaurants [3]. However, concerns about their health implications persist, as frankfurters are often associated with high levels of fat, sodium, and chemical preservatives [4,5]. In recent years, ethical, environmental, and nutritional concerns have driven increasing interest in the production of hybrid meat products, where lean meat is partially replaced by alternatives such as vegetables or edible insects [6,7,8]. Furthermore, dietary guidelines often recommend reducing meat consumption while encouraging a greater intake of plant-based foods [9]. Additionally, livestock production is associated with significant natural resource demand, including water, energy, and land, and contributes substantially to greenhouse gas emissions [10]. Consequently, alternative protein sources, such as plant-based options, are considered more sustainable compared to conventional animal protein.

In this context, consumers are increasingly seeking healthy and more sustainable meat products. This demand has driven the meat industry to explore alternative strategies for reformulating meat products to enhance their health appeal and overall attractiveness. The incorporation of vegetable components as meat replacements offers a promising approach to improving the nutritional value, safety, and shelf life of meat products. Previous studies have demonstrated positive outcomes by incorporating spinach, tomato, Barhi Date fruit pulp, plum, and lemon albedo powder into various meat products, including chicken, beef, and camel patties, sausages, hams, and turkey breast rolls [11,12,13,14,15]. These interventions were primarily aimed at reducing fat content, extending shelf life, and enhancing nutritional profiles. Additionally, plant-based ingredients such as chickpea, soybean flour, and textured soy protein have been used as partial meat substitutes in camel, beef, and chicken patties [6,7,16]. Other examples include the use of Chinese yam and arrowroot in hybrid chicken meat emulsions [17]. Despite these advancements, further exploration of plant-based ingredients remains essential to fully leverage their benefits in meat product development.

Tamarind (*Tamarindus indica* L.) is recognized as one of Brazil’s most significant tropical fruits. Although native to Madagascar, it is widely cultivated across Asia, Africa, and the Americas [18]. The pulp, which constitutes 30–50% of the tamarind pod, is the most consumed part of the plant. Rich in vitamin C and minerals, tamarind pulp serves as a souring agent in curries, sauces, and beverages, and is highly regarded in the culinary traditions of many countries [18]. Additionally, tamarind juice has been used as a natural beef tenderizer [19], while tamarind pulp paste has been incorporated into marination solutions to reduce the formation of polycyclic aromatic hydrocarbons during the grilling of pork meat [20].

However, the industrial processing of tamarind generates by-products, such as peels and seeds, which are often underutilized and discarded by both household and processing industries [18,21]. In this context, incorporating these by-products into food products represents a sustainable practice that can reduce tamarind waste generation while promoting recycling and reuse initiatives. These by-products are rich in bioactive compounds, including phenolic compounds, fatty acids, proteins, and minerals [22,23]. Therefore, exploring the potential use of tamarind by-products in meat products is of great importance. Tamarind seeds have already been incorporated into various food products, such as mango juice [24], noodles [25], bread, extruded snacks, and cookies [26], where they have contributed to an increase in protein content as well as enhanced antioxidant and functional properties. Additionally, tamarind peel has been used as a functional ingredient in cookies [27].

Due to their beneficial characteristics and properties, tamarind pulp paste, and its by-products can be easily incorporated into bakery products [28]. However, their application in meat products, such as frankfurter sausages, warrants further investigation. To the best of our knowledge, the direct use of tamarind pulp paste and its by-products in frankfurter sausages has not been explored. Therefore, the objective of this study was to investigate the impact of incorporating tamarind pulp paste, seeds, and peel as meat replacements on the nutritional, technological, structural, microbiological, and sensory characteristics of frankfurters.

## 2. Materials and Methods

### 2.1. Raw Materials

The tamarind pods used in this study were produced in Thailand and purchased at a local supermarket in Madrid, Spain, between October and November 2023. The lean pork meat, a blend of *M. biceps femoris*, *M. semimembranosus*, *M. semitendinosus*, *M. gracilis*, and *M. adductor*, contained 22.1 g/100 g protein and 5.1 g/100 g fat. Pork backfat, with 7.5 g/100 g protein and 86.7 g/100 g fat, was obtained from a local meat supplier in Madrid, Spain. The meat and the pork backfat were ground using a 6 mm plate, vacuum-sealed, and stored at −20 °C until further processing. The other ingredients of frankfurters comprised seasonings (Gewürzmüller, Salzburg, Austria), sodium chloride, sodium tripolyphosphate, and sodium nitrite (Panreac Química S.A., Barcelona, Spain).

### 2.2. Preparation of Tamarind Components

**Figure 1:**
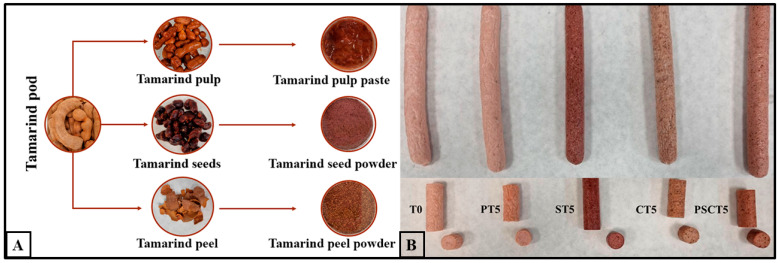
Typical appearance of tamarind and its components (**A**) and frankfurters elaborated with tamarind components (**B**). Treatments: T0 = control, frankfurters with no meat replacement; PT5 = frankfurters with 5% tamarind pulp paste addition as a meat replacer; ST5 = frankfurters with 5% tamarind seed addition as a meat replacer; CT5 = frankfurters with 5% tamarind peel addition as a meat replacer; PSCT5 = frankfurters with 5% of a tamarind pulp paste, seed and peel blend as a meat replacer.

To prepare tamarind pulp paste, the tamarind pods were cleaned and peeled. The pulp was soaked in water at a 1:1 (*w*/*v*) ratio and stored at 4 °C for 24 h, protected from light. After this period, the water was drained, the pulp was manually separated from the seeds, completely homogenized in a mixer (Infinyforce Pro Type DD95, Moulinex, Mayenne, France), and then frozen until use. Tamarind seeds were dried at 100 °C for 24 h, grounded (KG210, De’Longhi, Treviso, Italy), and frozen (−18 °C). The peel was obtained by grounding (KG210, De’Longhi, Treviso, Italy) until it formed a powder. Visual appearance regarding the tamarind and tamarind components is shown in Figure 1A.

### 2.3. Frankfurter Manufacturing

Five different frankfurter formulations were produced in duplicate at a pilot plant. The control formulation (T0) consisted of lean pork meat (60 g/100 g), pork backfat (19 g/100 g), water/ice (18.5 g/100 g), sodium chloride (1.65 g/100 g), seasoning (0.5 g/100 g), sodium tripolyphosphate (0.3 g/100 g), and sodium nitrite (0.05 g/100 g). The remaining four treatments contained the same ingredients as T0, with the addition of 5 g/100 g of tamarind pulp paste (PT5), tamarind seed powder (ST5), tamarind peel powder (CT5), or a blend of tamarind pulp paste, seed, and peel in a 1:1:1 ratio (PSCT5), replacing an equivalent amount of lean pork meat. The optimal inclusion levels of tamarind components were determined based on preliminary laboratory tests. As described by [29], the preparation method for the frankfurters began with thawing frozen meat and backfat at 3 ± 1 °C for 18 h. The raw meat and non-meat ingredients, added at various stages, were homogenized using a Thermomix^®^ TM6-1 (Vorwerk Elektrowerke GmbH & Co., Wuppertal, Germany), ensuring that the final temperature of all treatments remained below 10 °C. The resulting emulsion was then stuffed into 22 mm diameter cellulose casings (Viskase S.A., Lombard, IL, USA) and cooked in a steam oven (Combi-Master CM-6, Rational, Landsberg am Lech, Germany) at 80 °C and 99% relative humidity (RH) for 60 min. After cooking, the frankfurters were cooled with a water/ice mixture, chilled overnight, and the casings were removed the following day. The samples were then stored in a cooling chamber at 4 °C. Visual details of the frankfurters and their final appearance are presented in Figure 1B.

### 2.4. Composition

#### 2.4.1. Proximate Analysis

The proximate composition of frankfurter sausages was measured in triplicate according to the previous methods for moisture (950.46) and ash (920.153) [30]. The protein content was measured using a LECO FP-200 Nitrogen Determinator (Leco Corp., St. Joseph, MI, USA), while the fat content was evaluated as described by [31].

#### 2.4.2. Mineral Content

Mineral content was determined by preparing tamarind components and frankfurters through acid digestion with nitric acid in a microwave digestion system (ETHOS 1, Milestone S.r.l., Sorisole, Italy), as previously described by [32]. Mineral concentrations were measured using a ContrAA 700 High-Resolution Continuum Source spectrophotometer (Analytik Jena AG, Jena, Germany), equipped with a xenon short-arc lamp (GLE, Berlin, Germany). Three measurements were performed per sample to quantify the contents of calcium (Ca), magnesium (Mg), sodium (Na), potassium (K), phosphorus (P), zinc (Zn), and iron (Fe). The results were expressed as mg/100 g of the product.

### 2.5. Technological Properties of Frankfurters

#### 2.5.1. Processing Loss

Processing loss was calculated according to [33] by determining the weight loss (expressed as a percentage of the initial sample weight) of four frankfurters per treatment after heat processing and overnight chilling at 4 °C.

#### 2.5.2. pH

The pH of tamarind components and frankfurters was measured in triplicate using a digital pH meter (model 827, pH Lab Methrom, Herisau, Switzerland). Next, 10 g homogenized samples were dissolved in 90 mL of distilled water, and the pH was determined at 25 °C.

#### 2.5.3. Instrumental Color

CIELAB parameters (L*: lightness, a*: redness, and b*: yellowness) were measured on 2 cm thick slices of frankfurters (with nine measurements per sample) and across all tamarind components using a portable colorimeter (Konica Minolta CR-400, Tokyo, Japan). Measurements were taken under a D65 illuminant and a 10° standard observer with an 8 mm aperture, following the guidelines of the American Meat Science Association [34]. The color parameters of the tamarind components were specifically evaluated by placing the sample in transparent Petri dishes before measurement with the same colorimeter.

#### 2.5.4. Determination of Instrumental Texture

Textural properties of frankfurters were analyzed five times, using texture profile analysis (TPA) on a TA-XT.plus Texture Analyzer (Texture Technologies Corp., Scarsdale, NY, USA). The samples were cut into 2 cm pieces, and their hardness (N), cohesiveness (dimensionless), springiness (mm), and chewiness (N × mm) were measured. Textural parameters were determined by applying a double compression cycle test, compressing to 40% at a speed of 0.8 mm/s using a 5 kg load cell [35].

### 2.6. Structural Characteristics

Infrared spectra for each sample (frankfurters and tamarind components) were recorded using a Perkin-Elmer Spectrum™ 400 spectrometer (Perkin-Elmer Inc., Barcelona, Spain) in mid-infrared mode, equipped with an ATR sampling device. Approximately 25 mg of each sample (without prior preparation) was analyzed, with nine measurements per sample. Three sum spectra (72 accumulations) were recorded for each type of frankfurter. The spectral region from 2980 to 2820 cm^−1^ was examined to assess the lipid structure, as there is no interference from the tamarind peel in this region. To minimize interference from other compounds, primarily water, proteins, and tamarind seed, the 2125 cm^−1^ band of water, the amide II band around 1545 cm^−1^ from proteins, and the 1438 cm^−1^ band from tamarind seed were subtracted. The frequency and half-bandwidths of the _νas_CH2 (2918 cm^−1^) and _νs_CH2 (2851 cm^−1^) bands were measured in the resulting difference spectra [35,36].

### 2.7. Microbiological Analysis

A 10 g sample of each frankfurter treatment was aseptically transferred to a stomacher bag and homogenized with 90 mL of sterile 0.1% peptone water (Panreac, Darmstadt, Germany), and then a serial dilution was made. To determine the total viable counts (TVC), Plate Count Agar (PCA; Panreac) was utilized and incubated at 37 °C for 48 h. Enumeration of the Enterobacteriaceae was performed using Violet Red Bile Glucose Agar (VRBG; Panreac) with a double layer and incubated at 37 °C for 24 h. The count of mold and yeasts was performed using the Sabourad-Cloranfenicol Agar (SCA, Scharlab, Barcelona, Spain). Microbiological analyses were conducted in duplicate, and microbial counts were expressed as logarithms of colony-forming units per gram (log CFU/g) [37].

### 2.8. Sensory Analysis

Sensory evaluation of the frankfurters was conducted 24 h after processing by semi-trained panelists who were familiar with the product and had prior experience in sensory evaluations. The frankfurters were cut into 2.5 cm long pieces, randomly coded with a three-digit number, heated for 15 s in a microwave, and then immediately presented to the panelists. Water and unsalted breadsticks were provided to cleanse the palate between samples. A hedonic scale rating test was used, where panelists evaluated the appearance, color, texture, taste, aroma, general acceptability, and purchase intention of the frankfurters. The evaluation was performed on a 10 cm structured line scale, with “I completely dislike” and “I like very much” at the extremes. The purchase intention test also employed a 10 cm structured line scale, with “I definitely would not buy” and “I definitely would buy” at the endpoints. Each point was subsequently converted to a numerical value.

### 2.9. Statistical Analysis

The entire experiment was repeated twice on different days. A one-way analysis of variance was conducted using SPSS 26.0 software (SPSS Inc., Chicago, IL, USA) to analyze the effect of different tamarind parts as meat replacements on frankfurters. A completely randomized design included treatments (T0, PT5, ST5, CT5, and PSCT5) as fixed effects and two replications as random effects. Mean values were compared using Tukey’s HSD test, and differences were considered significant when *p* < 0.05.

## 3. Results and Discussion

### 3.1. Composition and Characterization of Frankfurters

#### 3.1.1. Proximate Composition

The proximate composition of the frankfurters with the addition of tamarind pulp paste and its by-products as meat replacements is presented in Table 1. The incorporation of tamarind components as substitutes for lean meat significantly affected (*p* < 0.05) the proximate composition of the frankfurters (Table 1). The moisture content ranged from 53.62 to 57.46 g/100 g, with no significant differences (*p* > 0.05) in moisture between the ST5 and PSCT5 treatments compared to the T0. However, the CT5 treatment exhibited the lowest moisture content (*p* < 0.05). Tamarind components with a high moisture content (pulp) did not produce frankfurters with higher moisture levels when substituting 5% of the meat. Thus, moisture values may be more related to emulsion stability and processing losses than to the composition of the meat substitute [17]. Regarding protein, the T0 treatment exhibited the highest protein content (16.23 g/100 g). As expected, a lower protein content was observed in all samples with tamarind compounds compared to control (Table 1) since they replace lean meat, the primary protein source in frankfurters. However, although statistically different, the protein content in CT5, ST5, and T0 was relatively comparable. Fat content ranged from 20.40 to 24.75 g/100 g, with PT5 showing the highest value (*p* < 0.05). Treatments incorporating tamarind pulp paste and its by-products generally exhibited higher fat content than the T0 treatment, with PT5 and CT5 having the highest fat values (Table 1). These results correlate with the lower (*p* < 0.05) moisture content, which may lead to the concentration of the remaining components in the frankfurters. Although tamarind components typically have a low fat content (between 1 and 2 g/100 g) [38], their inclusion may affect the overall fat distribution. Regarding ash content, T0 sausages exhibited significantly lower (*p* < 0.05) values compared to ST5, CT5, and PSCT5, which can be attributed to the higher mineral content of these tamarind components. This observation is supported by the mineral composition results (Table 2). The moisture, protein, fat, and ash in our study align with other studies on frankfurter reformulation with arbutus berries and rose hips [39].

#### 3.1.2. Mineral Content

The mineral content of the tamarind components and the frankfurters is presented in Table 2. Sodium, potassium, and phosphorus were the most prevalent minerals in all frankfurters, regardless of the tamarind component. In general, there were no significant differences in Na, K, and P content among the different sausages, except for the P content in CT5 (Table 2). Moreover, the addition of tamarind pulp paste and its by-products as meat replacements did not significantly influence these mineral levels. Sodium content ranged from 743.22 to 842.92 mg/100 g, with no significant differences (*p* > 0.05) among the treatments. These values were attributed to the direct addition of sodium chloride and nitrite during processing. The potassium content ranged from 172.07 mg/100 g to 202.08 mg/100 g. However, the sodium and potassium content reported in our study was similar to those reported by [8] in frankfurters. The incorporation of tamarind components resulted in a significant increase in Ca and Mg compared to the control, with the highest Ca values observed in CT5, as the peel contains the highest levels of Ca. In contrast, no differences in Mg content were observed based on the type of tamarind component incorporated (Table 2). In this context, the CT5 treatment can be claimed as “high in calcium” according to the European Regulation (EC No 1924/2006) [40]. Meat products with a high calcium content can be beneficial for human health because their consumption inhibits lipogenesis, regulates appetite, and reduces fat absorption in the gastrointestinal tract [41]. The Fe content decreased significantly in samples made with pulp, peel, and seed (Table 2) compared to the control, due to the 5% reduction in the meat content, which is the primary source of Fe in sausages. Regarding Zn, the primary source of this mineral in frankfurters is lean pork meat, which explains why the values observed for T0 were higher than those of the other treatments. Furthermore, the Zn levels are consistent with those reported for pork meat [42]. In addition, when incorporated into noodles, the tamarind components have already improved the calcium, magnesium, phosphorus, potassium, and sodium contents [25].

### 3.2. Technological Properties

#### 3.2.1. Processing Loss

**Figure 2:**
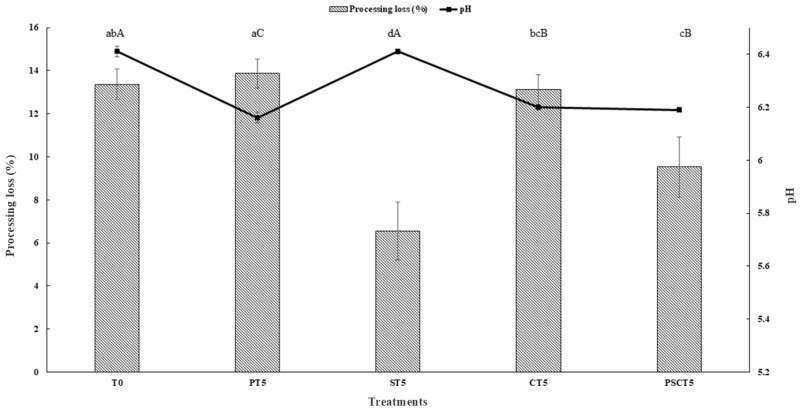
Processing loss and pH of frankfurters. Different superscript letters in rows (a–d) indicate statistically significant differences (*p* < 0.05) in the processing loss parameters. Different uppercase letters in rows (A–C) indicate statistically significant differences (*p* < 0.05) in the pH parameter. Treatments: T0 = control, frankfurters with no meat replacement; PT5 = frankfurters with 5% tamarind pulp paste addition as a meat replacer; ST5 = frankfurters with 5% tamarind seed addition as a meat replacer; CT5 = frankfurters with 5% tamarind peel addition as a meat replacer; PSCT5 = frankfurters with 5% of a tamarind pulp paste, seed, and peel blend as a meat replacer.

The processing loss is a crucial indicator closely tied to frankfurters’ water-holding capacity or emulsion stability/gel forming during cooking [8]. The cooking losses of frankfurters reformulated with tamarind components are shown in Figure 2. The partial replacement of lean pork meat with tamarind seeds (ST5) exerted a positive effect (*p* < 0.05) on the processing loss of frankfurters, as the reformulated samples showed a lower (*p* < 0.05) processing loss than T0. These findings can be attributed to the protein content in the seeds and their gelling properties, which improve the emulsion stability [43]. Nonetheless, our findings align with those reported in frankfurters with other seeds such as the addition of chia and sunflower seed flour [44,45].

#### 3.2.2. pH

The pH values of the frankfurters ranged from 6.16 to 6.41 (Figure 2) and were influenced (*p* < 0.05) by the different components of tamarind. The lowest (*p* < 0.05) pH value was observed in the PT5 treatment, while the highest (*p* < 0.05) was reported for the T0 and ST5 treatments. The lower pH in PT5 is likely due to the acidic nature of the tamarind pulp paste (pH 3.45 ± 0.02 ^c^) used, whereas the seeds and peel exhibited high pH values (5.44 ± 0.01 ^a^ and 4.40 ± 0.02 ^b^, respectively). A similar reduction in pH values has been reported in grilled pork marinated with tamarind pulp paste [20]. Other studies also related lower pH values in frankfurters with the addition of banana by-products and tomato powder [11,46].

#### 3.2.3. Determination of Instrumental Color

**Figure 3:**
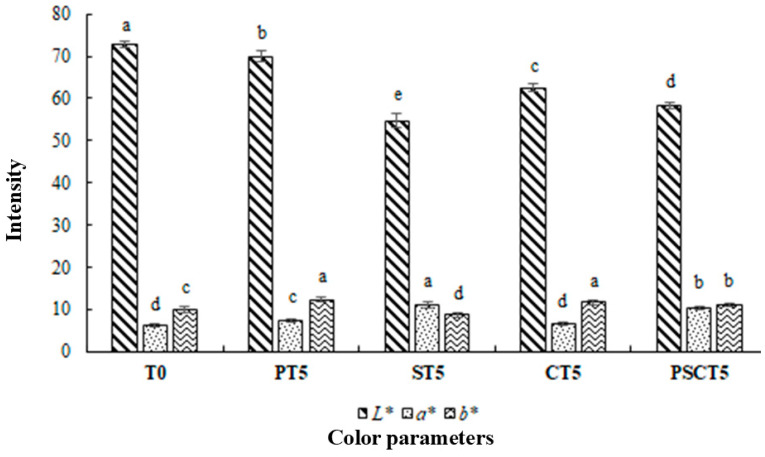
Color parameters (L*, a* and b*) of frankfurters. Different superscript letters (a–e) indicate statistically significant differences (*p* < 0.05) for each color parameter. Treatments: T0 = control, frankfurters with no meat replacement; PT5 = frankfurters with 5% tamarind pulp paste addition as a meat replacer; ST5 = frankfurters with 5% tamarind seed addition as a meat replacer; CT5 = frankfurters with 5% tamarind peel addition as a meat replacer; PSCT5 = frankfurters with 5% of a tamarind pulp paste, seed, and peel blend as a meat.

Color has a significant role in meat and meat products, such as frankfurters, because it is directly related to consumer acceptance [47]. The color parameters (L*, a*, and b* values) of each frankfurter treatment are shown in Figure 3. It can be concluded that the addition of tamarind pulp paste, seeds, peel, and their blend at 5% to replace lean pork meat significantly reduced (*p* < 0.05) the luminosity of the frankfurter sausages, resulting in darker products. Significant differences were observed in the L* values across all treatments. The T0 treatment exhibited the highest L* value (72.78), while the ST5 treatment showed the lowest lightness value (54.72) (*p* < 0.05). Regarding a* values, no statistical difference was observed between T0 and CT5 (*p* > 0.05), both showing the lowest values for this parameter (6.31 and 6.57, respectively). In comparison to T0, the PT5, ST5, and PSCT5 treatments showed a higher a* content (*p* < 0.05) (Figure 3), with ST5 showing the highest a* value (11.03), indicating a more intense red color than the other treatments. These results for L* and a* values are consistent with the direct visual observation of the reformulated sausages with the different tamarind components (Figure 1). These color alterations among treatments are due to tamarind compounds’ darker and reddish color (except the peel) than the lean pork meat. ST5 showed the lowest values of b* (8.68) (*p* < 0.05), while PT5 and CT5 presented the highest values of this color parameter (*p* > 0.05). Although the addition of tamarind components (such as pulp paste, seeds, peels, or their blend) did not significantly alter all color parameters of the frankfurters, the final color and appearance of meat products can generally be influenced by the inclusion of these ingredients. When new ingredients are added to meat products, the color may be affected by both the type of ingredient and its concentration, especially when used to replace meat [48]. A similar trend has been observed in low-fat and low-salt frankfurters enriched with mushroom flours (*Agaricus bisporus* and *Pleurotus ostreatus*), where the L* and a* color parameters varied depending on the concentration and type of mushroom incorporated into the formulation [49].

#### 3.2.4. Determination of Texture Parameters

Texture parameters are also crucial for determining the sensory properties and acceptance of meat products [50]. Replacing lean pork meat with tamarind components resulted in significant changes in the hardness, cohesiveness, springiness, and chewiness of the frankfurters (Table 3). The incorporation of 5% tamarind seeds (ST5), peel (CT5), and their combination (PSCT5) resulted in an increase (*p* < 0.05) in the hardness and chewiness of the frankfurters, with the most significant effect observed in the frankfurters containing tamarind seeds (ST5) (Table 3). The firmer texture in ST5 can be attributed to the higher protein content of tamarind seeds compared to the other tamarind components, as well as the physical characteristics of the seeds that remain after grinding. This effect is so pronounced that, even when the seeds were mixed with other tamarind components, the PSCT5 treatment exhibited greater (*p* < 0.05) hardness than the T0, PT5, and CT5 treatments.

In contrast, the inclusion of 5% pulp paste (PT5) did not result in any significant differences (*p* > 0.05) in hardness compared to T0. Regarding cohesiveness, the ST5 treatment exhibited lower values (*p* < 0.05) than T0 and the other frankfurter treatments (PT5, CT5, and PSCT5). Similarly, the addition of mushroom flours to frankfurters led to decreased TPA parameter values [49]. However, the inclusion of chia seeds in frankfurter formulations has been reported to result in a final product with lower hardness values compared to the control [51]. This contrasts with our findings, where the addition of tamarind seeds (ST5) led to an increase in hardness.

### 3.3. Structural Characteristics

The spectral region comprised an area between 2950 and 2830 cm^−1^ (acyl chain region); this permits us to study the lipid structure, including lipid–protein interactions [52]. In this spectral region, the analysis of the half-bandwidths of the νasCH2 (2918 cm^−1^) and (νsCH2) (2851 cm^−1^) bands from the CH2 group in alkyl lipid chains are conformation-change-sensitive [52]. Table 3 show that non-significant changes in half-bandwidth of the 2918 cm^−1^ (νasCH2) and 2851 cm^−1^ (νsCH2) bands were observed when we compared the different frankfurters. These results could be associated with a lack of variation in the conformational order of the lipid acyl chains and their dynamics [52], due to the incorporation of tamarind compound. This could be attributed to a lack of chemical interaction between these compounds and lipids and, consequently, the absence of changes in the interactions between these lipids and the rest of the components of the frankfurters, such as proteins. This behavior is in consonance with other studies in which no structural changes in proteins have been observed due to the incorporation of tamarind seed powder into gelled food matrices [53]. The physical entrapment of tamarind compounds within the frankfurter matrix appears to be linked to their distinct textural behavior (Table 3), which, in turn, seems to depend on various factors, including the specific physical characteristics of each tamarind component (Figure 1).

### 3.4. Microbiological Properties

**Figure 4:**
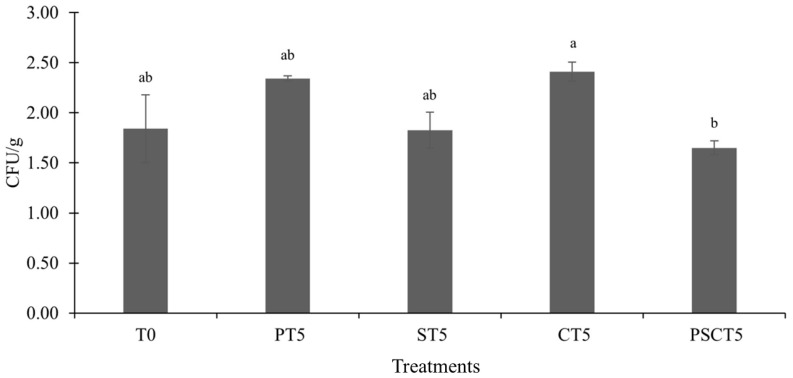
Total viable counts (TVC) (CFU/g sample) of frankfurters. Different superscript letters in rows (a–b) indicate statistically significant differences (*p* < 0.05). Treatments: T0 = control, frankfurters with no meat replacement; PT5 = frankfurters with 5% tamarind pulp paste addition as a meat replacer; ST5 = frankfurters with 5% tamarind seed addition as a meat replacer; CT5 = frankfurters with 5% tamarind peel addition as a meat replacer; PSCT5 = frankfurters with 5% of a tamarind pulp paste, seed, and peel blend as a meat replacer.

The microbiological quality of frankfurters is generally influenced by their composition, particularly when new ingredients or by-products are introduced. However, in this study, no significant differences were observed in the microbiological counts of frankfurters with the addition of tamarind components, and the microbial levels remained low. The TVC on day one of storage ranged from 1.65 log CFU/g to 2.41 log CFU/g, and the CT5 treatment had higher counts (*p* < 0.05) than PSTC5 (Figure 4). The other tamarind components do not appear to affect the TVC. No differences in TVC were observed between T0 and the treatments with the addition of tamarind components. These initial counts were very low and unlikely to cause any sensory degradation of products, such as off-flavors [51]. Overall, the incorporation of tamarind did not affect microbial growth, and the products were as safe as the control. Additionally, *Enterobacteriaceae*, molds, and yeasts were not detected, further emphasizing the good microbiological quality of the frankfurters prepared with tamarind components.

### 3.5. Sensory Analysis

**Figure 5:**
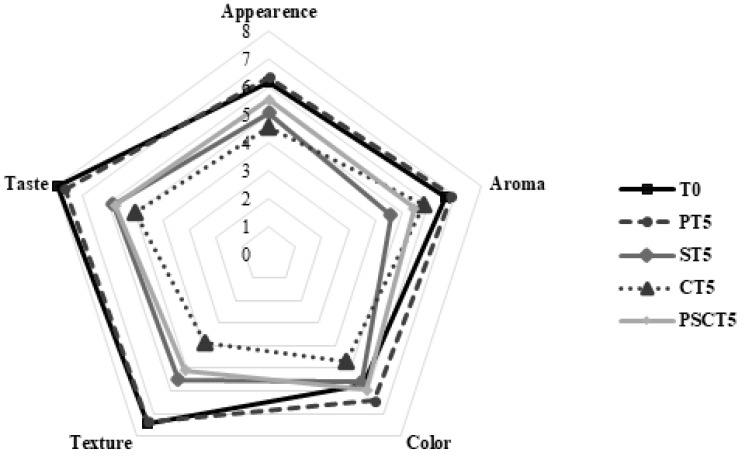
Sensory evaluation of frankfurters. Treatments: T0 = control, frankfurters with no meat replacement; PT5 = frankfurters with 5% tamarind pulp paste addition as a meat replacer; ST5 = frankfurters with 5% tamarind seed addition as a meat replacer; CT5 = frankfurters with 5% tamarind peel addition as a meat replacer; PSCT5 = frankfurters with 5% of a tamarind pulp paste, seed, and peel blend as a meat replacer.

Figure 5 presents the average scores for appearance, color, texture, taste, and odor of frankfurters with the addition of tamarind components and their blend. No statistical difference was observed in the appearance parameter among the samples; however, the PT5 treatment exhibited the highest (*p* < 0.05) score (6.37) for this attribute, followed by T0 (6.21). Regarding odor, a statistical difference was only observed between PT5 and ST5, with scores of 6.85 and 4.59, respectively, while the other treatments were statistically similar to each other. No statistical difference was detected in terms of color, although the sausages’ visual appearance varied, as shown in Figure 1B. The sensory analysis of texture indicated that treatments T0 and PT5 were similar (*p* > 0.05). In contrast, treatments CT5 and PSCT5 exhibited similar (*p* > 0.05) texture scores to each other but were significantly lower than those of T0 and PT5. These results also correlate with our TPA findings, where frankfurters with lower hardness values in TPA (T0 and PT5) were rated more positively in the sensory panel, indicating a possible consumer preference for softer, less firm frankfurters.

Regarding overall acceptance, T0 and PT5 were statistically similar (*p* > 0.05) and received scores above 7, while CT5 had the lowest score (4.11) which was statistically similar to ST5 and PSCT5. For purchase intention, T0 and PT5 received scores of 7.15 and 7.25, respectively, whereas CT5 had the lowest score (3.54) (*p* < 0.05).

Furthermore, PT5 showed scores higher than 7 for purchase intention, suggesting that the addition of 5% tamarind pulp paste was better accepted than other tamarind components (Figure 5). The higher sensory attribute scores in the PT5 treatment could be linked to consumer expectations for frankfurter sausages, as this treatment was the most similar to T0, also evident in the direct visual observation (Figure 1B). The lower scores for sensory attributes in the ST5, CT5, and PSCT5 treatments (all under 6.0) may be attributed to some panelists noting unpleasant texture sensations, likely due to the use of dry tamarind seed or peel. Future studies should explore incorporating tamarind seed and peel in frankfurter sausages in different forms to enhance acceptability.

## 4. Conclusions

The reformulation of frankfurters with tamarind pulp paste, seeds, peel, and their blend as lean pork meat replacements resulted in notable changes in the nutritional, technological, microbiological, and sensory characteristics of these products. Despite differences observed in the characterization analysis, using different tamarind components proved viable. Furthermore, incorporating tamarind components into frankfurters demonstrated sustainability and innovation, offering a practical solution for repurposing tamarind processing by-products, which are typically considered waste. Among the formulations tested, tamarind pulp paste (PT5) emerged as the most effective meat alternative for frankfurter sausages. This reformulation preserves product quality and promotes the sustainability of frankfurter production, significantly contributing to more environmentally friendly meat processing practices. Future studies will further investigate the impact of tamarind components on the shelf-life of frankfurter sausages.

## Figures and Tables

**Table 1:** Proximal composition (%) of frankfurters.

	Treatments				
	T0	PT5	ST5	CT5	PSCT5
Moisture	57.46 ± 0.22 ^a^	55.74 ± 0.21 ^b^	56.76 ± 0.44 ^a^	53.62 ± 0.45 ^c^	57.19 ± 0.30 ^a^
Protein	16.23 ± 0.30 ^a^	13.75 ± 0.17 ^c^	15.13 ± 0.34 ^b^	15.30 ± 0.36 ^b^	14.03 ± 0.11 ^c^
Fat	20.40 ± 0.61 ^d^	24.75 ± 0.18 ^a^	21.31 ± 0.12 ^c^	23.36 ± 0.14 ^b^	22.77 ± 0.35 ^b^
Ash	2.44 ± 0.03 ^c^	2.51 ± 0.01 ^bc^	2.52 ± 0.04 ^b^	2.59 ± 0.00 ^a^	2.54 ± 0.01 ^ab^

Mean ± SD. Different superscript letters in rows (^a–d^) indicate statistically significant differences (*p* < 0.05). Treatments: T0 = control, frankfurters with no meat replacement; PT5 = frankfurters with 5% tamarind pulp paste addition as a meat replacer; ST5 = frankfurters with 5% tamarind seed addition as a meat replacer; CT5 = frankfurters with 5% tamarind peel addition as a meat replacer; PSCT5 = frankfurters with 5% of a tamarind pulp paste, seed, and peel blend as a meat replacer.

**Table 2:** Mineral composition (mg/100 g) of frankfurter sausages and tamarind components.

	Treatments					Tamarind Components		
	T0	PT5	ST5	CT5	PSCT5	Pulp Paste	Seeds	Peel
Ca	12.85 ± 0.66 ^bc^	9.09 ± 0.30 ^c^	15.78 ± 1.17 ^bc^	68.73 ± 8.20 ^a^	26.53 ± 4.92 ^b^	55.77 ± 5.26 ^z^	98.14 ± 3.05 ^y^	714.25 ± 15.42 ^x^
Mg	21.6 ± 0.13 ^b^	20.20 ± 0.83 ^b^	25.41 ± 0.04 ^a^	25.85 ± 0.89 ^a^	25.12 ± 0.74 ^a^	45.37 ± 1.36 ^z^	162.60 ± 5.49 ^x^	100.04 ± 1.39 ^y^
Na	801.15 ± 36.24 ^a^	743.22 ± 26.76 ^a^	842.92 ± 51.69 ^a^	777.95 ± 50.80 ^a^	795.59 ± 15.22 ^a^	0.47 ± 0.23 ^x^	0.34 ± 0.02 ^x^	0.51 ± 0.06 ^x^
K	192.48 ± 10.86 ^a^	181.62 ± 1.03 ^a^	172.07 ± 4.69 ^a^	189.90 ± 11.24 ^a^	202.08 ± 7.95 ^a^	806.31 ± 1.64 ^x^	715.51 ± 33.57 ^x^	800.75 ± 131.48 ^x^
P	200.13 ± 13.06 ^a^	177.73 ± 12.28 ^ab^	177.70 ± 4.48 ^ab^	162.37 ± 3.82 ^b^	185.80 ± 3.70 ^ab^	17.62 ± 1.48 ^y^	86.50 ± 3.49 ^x^	26.33 ± 5.91 ^y^
Zn	1.79 ± 0.06 ^a^	1.67 ± 0.04 ^ab^	1.55 ± 0.09 ^bc^	1.40 ± 0.01 ^c^	1.50 ± 0.06 ^bc^	0.72 ± 0.03 ^y^	3.16 ± 0.15 ^x^	0.91 ± 0.03 ^y^
Fe	0.92 ± 0.02 ^a^	0.69 ± 0.02 ^b^	0.68 ± 0.01 ^b^	0.66 ± 0.00 ^b^	0.88 ± 0.01 ^a^	0.40 ± 0.01 ^z^	2.14 ± 0.06 ^x^	0.98 ± 0.03 ^y^

Mean ± SD. Different superscript letters in rows (^a–c^) indicate statistically significant differences (*p* < 0.05) in frankfurter treatments. Different superscript letters in rows (^x–z^) indicate statistically significant differences (*p* < 0.05) in tamarind components. Treatments: T0 = control, frankfurters with no meat replacement; PT5 = frankfurters with 5% tamarind pulp paste addition as a meat replacer; ST5 = frankfurters with 5% tamarind seed addition as a meat replacer; CT5 = frankfurters with 5% tamarind peel addition as a meat replacer; PSCT5 = frankfurters with 5% of a tamarind pulp paste, seed, and peel blend as a meat replacer.

**Table 3:** Texture profile analysis (TPA) parameters and half-bandwidth values of the 2918 cm^−1^ (ν_as_CH_2_) and 2851 cm^−1^ (ν_s_CH_2_) bands of frankfurters.

	Treatments				
	T0	PT5	ST5	CT5	PSCT5
Hardness (N)	13.08 ± 0.33 ^d^	13.68 ± 0.01 ^d^	38.50 ± 1.84 ^a^	19.52 ± 0.45 ^c^	23.73 ± 1.09 ^b^
Cohesiveness	0.65 ± 0.01 ^ab^	0.68 ± 0.01 ^a^	0.58 ± 0.02 ^c^	0.64 ± 0.01 ^b^	0.63 ± 0.01 ^b^
Springiness (mm)	7.41 ± 0.42 ^a^	7.55 ± 0.1 ^a^	7.86 ± 0.17 ^a^	7.24 ± 0.30 ^a^	7.86 ± 0.44 ^a^
Chewiness (N × mm)	63.13 ± 4.97 ^d^	69.84 ± 3.34 ^d^	174.69 ± 16.21 ^a^	90.51 ± 4.86 ^c^	118.42 ± 11.12 ^b^
Half-bandwidth 2918 cm^−1^	27.39 ± 0.4 ^a^	26.66 ± 0.7 ^a^	27.59 ± 0.5 ^a^	27.01 ± 0.6 ^a^	26.73 ± 0.8 ^a^
Half-bandwidth 2851 cm^−1^	15.72 ± 0.1 ^a^	15.70 ± 0.2 ^a^	15.61 ± 0.3 ^a^	15.41 ± 0.1 ^a^	15.36 ± 0.2 ^a^

Mean ± SD. Different superscript letters in rows (^a–d^) indicate statistically significant differences (*p* < 0.05). Treatments: T0 = control, frankfurters with no meat replacement; PT5 = frankfurters with 5% tamarind pulp paste addition as a meat replacer; ST5 = frankfurters with 5% tamarind seed addition as a meat replacer; CT5 = frankfurters with 5% tamarind peel addition as a meat replacer; PSCT5 = frankfurters with 5% of a tamarind pulp paste, seed, and peel blend as a meat replacer.

## Data Availability

The original contributions presented in this study are included in the article. Further inquiries can be directed to the corresponding authors.
